# Paraneoplastic cerebellar degeneration associated with ovarian cancer

**DOI:** 10.3892/ol.2012.1016

**Published:** 2012-11-07

**Authors:** ALESSIA ERIKA RUSSO, SIMONA SCALONE, GIULIA COSTANZA LEONARDI, AURORA SCALISI, GIORGIO GIORDA, ROBERTO SORIO

**Affiliations:** 1 Department of Biomedical Sciences, Section of Pathology and Oncology, Laboratory of Translational Oncology and Functional Genomics, University of Catania, Catania 95124;; 2 Department of Medical Oncology C, National Cancer Institute, IRCCS, Aviano 33081;; 3 Secondary Prevention and Screening Gynecological Unit, ASP, Catania 95100;; 4 Unit of Gynecologic Oncology, National Cancer Institute, IRCCS, Aviano 33081, Italy

**Keywords:** paraneoplastic cerebellar degeneration, anti-Yo, ovarian cancer

## Abstract

Paraneoplastic cerebellar degeneration (PCD) is a rare neurological disorder characterized by a widespread loss of Purkinje cells associated with a progressive pancerebellar dysfunction. PCD often precedes the cancer diagnosis by months to years. Here, we report the case of a 64-year-old woman who developed PCD symptoms, associated with high levels of anti-Yo antibodies, one year after a previous diagnosis of ovarian cancer. Clinical features, pathogenesis and treatment of PCD associated with cancer are discussed according to previous studies.

## Introduction

Paraneoplastic neurological syndromes (PNSs) are a heterogeneous group of neurologic disorders caused by an immune response to a primary malignancy. It has been revealed that PNS may precede the diagnosis of cancer in 50–80% of cases (1). The exact incidence of PNS among those diagnosed with cancer remains uncertain, with estimates ranging from 1 in 10,000 to 1 in 100. Paraneoplastic sensory neuropathy (PSN) is probably the most common type of PNS (accounting for 3-7/1000 cancer diagnoses), followed closely by paraneoplastic encephalitis (PEM) (3/1000) and paraneoplastic cerebellar degeneration (PCD) (2/1000) (2).

PNS is initiated as a peripheral immune response directed against autoantigens expressed in tumors. A cancer-stimulated immune reaction that crossreacts with neural tissue, i.e., onconeural immunity, is considered to be the principal pathological mechanism for PNS. The oncoantigens that drive the immune response are normally restricted to the nervous system (3). The neurological attack may affect the central, peripheral somatic or autonomic nervous systems and presentations are commonly multifocal rather than ‘classical’ syndromes.

PCD is a rare but fatal neuronal syndrome associated with ovarian, breast and lung cancer patients (4–8). It is characterized by cerebellar atrophy with a diffuse loss of Purkinje cells, mediated by a cross-reaction of antibodies with tumor antigens and cerebellar tissue (3,9–12). PCD-related autoantibodies include: i) anti-Hu, ii) anti-Ri/Nova and iii) anti-Yo. Anti-Hu and anti-Ri/Nova are detected in patients with small cell lung and breast cancer, respectively (13). Anti-Yo, also called Purkinje cell cytoplasmic antibody type 1 (PCA-1), is usually associated with ovarian and other gynecologic cancers (14,15). It is an immunoglobulin (Ig) G directed against the cytoplasmic antigen cerebellar degeneration-related protein 2 (CDR2), detected in the central nervous system and tumor tissue. Intracellular antigens are not accessible to immune attack *in situ*, but peptides derived from intracellular proteins are displayed on upregulated major histocompatibility complex (MHC) class-I molecules in a proinflammatory cytokine milieu following proteasomal degradation, and are then accessible to peptide-specific cytotoxic T cells. Antibodies targeting nuclear or cytoplasmic antigens are serum markers of T cell effector-mediated injury. PCA-1 targeting these intracellular antigens is detected in serum and cerebrospinal fluid (CSF), but is not directly involved in the pathogenesis of neural tissue damage. In clinical practice, these antibodies serve as diagnostic markers of a T cell predominant effector process. CDR2 displayed in upregulated MHC class-I molecules is then accessible to peptide-specific cytotoxic T cells. Emigration of expanded populations of MHC class-I-restricted molecules and CD8^+^ onconeural peptide-specific cytotoxic T lymphocytes from tumor-draining lymph nodes to the systemic circulation, and thence to the CNS, is a plausible mechanism for neuronal degeneration in patients with PCA-1 autoimmunity (16–18).

Clinical manifestations of PCD are usually characterized by subacute onset but progressive pancerebellar dysfunction, including truncal and appendicular ataxia, dysarthria, vertigo, nystagmus and diplopia (19). These symptoms progress over weeks to months and then stabilize, leaving the patient severely disabled. It has also been observed that PCD precedes tumor occurrence by months or even years (8,20–22). In this report, we describe a case of a 64-year-old female patient developing PCD one year after the diagnosis of ovarian cancer. The study was approved by the ethics committee of the National Cancer Institute, IRCCS of Aviano and informed consent was obtained from the patient’s family.

## Case report

In June 2008, a 64-year-old female patient presented to the Department of Medical Oncology C at the National Cancer Institute (Aviano, Italy) with a two-month history of abdominal distension and pelvic pain, and markedly elevated levels of CA-125. Abdominal computed tomography (CT) identified large, bilateral, irregular and inhomogeneous ovarian masses inseparable from the uterus, as well as massive ascites and several small, confluent pelvic lymph nodes consistent with metastases. Total abdominal hysterectomy and bilateral salpingo-oophorectomy, omentectomy, rectum-sigma resection, and bilateral pelvic and lombo-aortic lymphadenectomy were conducted. Histology revealed a high-grade ovarian serous papillary adenocarcinoma with rectal and appendicular involvement, as well as metastases in 23 out of 24 lymph nodes examined (FIGO stage IIIc). The patient achieved complete remission following six courses of treatment with paclitaxel (250 mg/m^2^) and carboplatin (AUC 5), and since then has remained disease-free.

One year later, in June 2009, the patient was admitted to a neurology clinic for subacute onset of dysmetria with truncal and appendicular ataxia, dysgraphia, nystagmus, diplopia and mild dysphagia for liquids. A brain MRI did not reveal any mass lesion or signs of cerebellar atrophy, stroke or cerebellitis. The electromyogram and CA-125 levels were normal. A total body contrast-enhanced CT scan was negative for ovarian cancer recurrence. The detection of a high titer of anti-Yo onconeural antibodies in the CSF and blood, confirmed the clinical suspicion of PCD. High doses of intravenous Ig (400 mg/kg daily for 5 days) were administered in conjunction with corticosteroids without significant clinical improvement. One month later, the patient was referred again to our department. The neurological symptoms were significantly worse and the patient was unable to walk or talk, and developed severe dysphagia to both solids and liquids. We arranged a whole-body PET/CT scan to check for disease relapse but no evidence of abnormal hot spots was noted. The patient received other three cycles of intravenous Ig every 4–6 weeks and monthly boluses of cyclophosphamide (800 mg/m^2^). No benefits were observed; therefore, a decision was made to terminate this immunosuppressive therapy. In October 2009, a new PET/CT scan was conducted. There was no evidence of recurrence, and a neurological follow-up at 3 months demonstrated stabilization in neurological status.

In June 2010, one year after the onset of neurological symptoms, the patient presented with an involuntary loss of fecal material and gas, as well as an intermittent discharge of mucus through the vagina. A CT scan of the abdomen, obtained two hours after administration of an oral contrast medium, revealed a fistulous communication between the vagina and a loop of sigmoid colon, as well as an accumulation of contrast material in the vaginal vault. For this purpose, the patient underwent laparoscopic surgery confirming the CT scan diagnosis. Due to the poor performance status, the patient was admitted to a Palliative and Supportive Care Unit.

## Discussion

PCD has been described prior to the appearance of a primary tumor (8,20–22). However, to our knowledge, only two cases of PCD have occurred in patients with a clinical history of cancer (23,24). In the present report, we described the case of a patient who developed PCD one year after a previous diagnosis of ovarian cancer. The patient presented with neurological symptoms and a high titer of anti-Yo antibodies in the CSF and peripheral venous blood.

In patients with ovarian cancer, only mild to moderate neurological improvements have been achieved following a combination of treatment, including plasmapheresis, intravenous Ig and chemotherapy, for the underlying neoplasm (25). There are no established protocols for the treatment of most paraneoplastic syndromes. The physician may employ either plasma exchange or a combination of intravenous Ig and immunosuppressive agents, including corticosteroids or cyclophosphamide. Although there have been occasional reports of improvement with these therapies, generally, there is a minimal effect since the antibodies are intrathecal and unaffected by plasmapheresis or intravenous Ig (3,25). Symptom relief is important in the management of patients with PCD. Intensive rehabilitation, speech therapy and psychological support are also vital in optimizing functional recovery (26). However, it has been demonstrated that early therapy of PCD may improve neurological status (27–32). Accordingly, we started immunomodulatory treatment regimes with high doses of intravenous Ig, corticosteroids and cyclophosphamide four weeks after the onset of PCD; however, our patient did not receive any benefit from this treatment. The failure of this therapy may be associated with neuronal loss, which was already high and irreversible prior to the diagnosis of PCD. A few cases with early diagnosis may have mild and reversible damage, thus responding favorably to treatment.

In conclusion, although the majority of cases of tumor occurrence are preceded months or even years by cerebellar signs, in this case report we observed that PCD may also emerge following the appearance of a primary tumor. Despite anecdotal case reports revealing neurological improvement with various combinations of treatment, there remains a requirement for greater efficacy in therapy for PCD. Further investigations on the pathogenesis of PCD are required to identify more effective therapies, which are able to stabilize or reverse the neurological symptoms.
